# Targeting the Psychosexual Challenges Faced by Couples with Breast Cancer: Can Couples Group Psychotherapy Help?

**DOI:** 10.4172/2167-0420.1000205

**Published:** 2014-11-17

**Authors:** Luciana Lagana, Patricia Fobair, David Spiegel

**Affiliations:** 1 Department of Psychology, California State University Northridge, California, USA; 2 Department of Psychiatry and Behavioral Science, Stanford University School of Medicine, USA

**Keywords:** Cancer, Breast cancer, Group psychotherapy, Sexual problems, Sexual dysfunction, Psychotherapeutic techniques, Intimacy, Couple relations, Sex therapy, Group therapy, Couples therapy

## Abstract

The need for the psychosexual rehabilitation of breast cancer survivors and their intimate partners is underscored by the high prevalence of multiple psychosexual difficulties encountered by this patient population. Concerns about health, sexuality, and emotional distress are common among women with breast cancer and are often related to the side effects of cancer treatment. Additionally, both intimate relationship problems and partners’ distress are likely to influence patients’ psychosexual health. A clearer understanding of these complex clinical issues is needed in order to implement effective psychosexual rehabilitation interventions. In this article, we extended the use of the manualized and empirically validated Supportive-Expressive Group Therapy (SEGT) model to target the specific psychosexual needs of couples with breast (as well as other types of) cancer. In view of the pertinent literature in this area and based on our clinical experience utilizing this group therapy model with different patient populations, we have discussed how clinicians involved in the psychosexual care of oncology patients could apply such a model within a couples group therapy format.

## Introduction

Due to the emotional significance of the breast and the related fear that its removal might be necessary for treatment, breast cancer is viewed as particularly terrifying, creating significant anxiety and distress even prior to diagnosis [1]. Women who undergo breast cancer treatment are at risk for persistent and significantly disrupted quality of life and emotional distress [2,3].

### The importance of the problems targeted in this review

Unfortunately, sexual dysfunction is a likely complication of breast cancer, often related to the various consequences of medical treatment, partner distress, and intimate relationship difficulties. Especially in the first months after medical treatment, sexual problems are very common, and they are significantly associated with body image difficulties, vaginal dryness, marital status, as well as partners’ difficulties understanding the patient’s feelings [4]. Women treated with either breast conserving modalities or with mastectomy report somewhat similar psychosexual and body image problems (usually loss of libido, loss of interest in partner, and sexual dissatisfaction). Because these problems surface early on, sexuality issues should be properly assessed and targeted therapeutically as soon as possible [5].

### The necessity of presenting this review

In the busy practice of oncologists, intimacy and sexuality are often forgotten and sidelined, as the focus is the disease and related multimodalities of therapy. Similarly, in research, the psychosexual problems of *couples* living with breast cancer are relatively unexplored, and this is particularly the case regarding group therapy options. The work presented herein is intended to encourage the health providers of breast cancer patients to consider offering more holistic care, given the importance of these neglected psychosexual issues.

To fill a gap in the literatureon models for couples group therapy with breast cancer patients and their intimate partners, we have made an effort to integrate a summary of the available pertinent literature (using the websites MEDLINE/PubMed and Psyc INFO) with clinical insights stemming from our own professional experience in the clinical field of couples group therapy. We have used the terms “spouse” and “intimate partner” interchangeably.

### Method

We have first summarized the literature on the psychosexual challenges faced by breast cancer survivors and their intimate partners, and then have provided clinical recommendations for health professionals interested in conducting group psychotherapy in a couples group therapy format. In the first place, the current *context* of breast cancer survivors must be considered. Several clinical decisions must be made, for instance, about what kind of “cancer patient and partner” couples could join such a therapy group, and whether it would be appropriate to have patients at different disease stages in the same group. We believe that it is up to the individual clinician to determine whether the couples groups will contain both asymptomatic primary cancer survivors and women with metastatic or terminal cancer. Some of the themes of the Supportive Expressive model discussed below could apply more to one set of patients or to the other, depending on the specific theme. For example, women with primary disease who underwent successful surgery are unlikely to be very concerned about death or symptom control, as these types of issues are likely to be more applicable to women with incurable cancer. However, the therapeutic interaction of women at different stages of the disease could generate several positive outcomes. For instance, asymptomatic women could normalize and hopefully accept more fully their cancer experience once they are exposed to the realities of survivors who underwent (or are still undergoing) more painful and prolonged treatment of the disease. Our clinical suggestions stem from the aforementioned review of the literature (published mainly within the past two decades) and from our experiences using our classic model (briefly described herein) when providing SEGT to medically ill patients through a manualized, empirically validated group therapy approach [6,7]. In particular, we have discussedhow each component of this therapeutic model could be utilized to address the psychosexual challenges of couples who are coping with a serious medical illness such as breast cancer. Given the innovativeness of using a couples group therapy approach with this medical patient population, at times we could not locate any relevant studies to cite, yet we still offered our preliminary insights on such understudied clinical issues. Involvement of the sexual partner/spouse of the patient could be particularly helpful, in view of findings such as those of Abasher [8], who identified length of marriage to be a protective factor for sexual dysfunction in a Sudanese sample of breast cancer patients undergoing hormonal treatment. The length of the marriage could reflect the depth of commitment and the potential openness of both partners to sharing life experiences that are intense such as having to face mortality. Thus, it makes sense to conceptualize couples group therapy as being potentially helpful for these patients. Moreover, there is some literature [9] showing that early stage breast cancer patients undergoing six sessions of couple-focused group interventions report lower levels of depressive symptomatology than patients assigned to six sessions of usual care. Therefore, it is possible that couples group is a neglected treatment modality with significant psychological as well as sexual benefits. Indeed, treating cancer patients’ depression is particularly important, given that its amelioration is related to longer survival in metastatic breast cancer patients [10].

It should be noted that most of the literature cited herein refers to psychosexual problems encountered by breast cancer survivors involved in a heterosexual relationship. However, a brief mention of the available literature in this area concerning lesbian populations is in order. In this regard, in a study conducted Fobair et al. [11], SEGT helped lesbian patients with breast cancer, as they reported better sleep and lower pain levels as well as decreased conflicts with their family members. However, no changes in sexuality or body image were found: this indicates the necessity to address sexual problems directly in order to see improvements in sexual functioning. Additionally, comparing lesbian and heterosexual women living with newly diagnosed breast cancer, Fobair et al. [12] found better body image among lesbians, but no significant differences in sexual activity. More treatment outcome research on this topic needs to be conducted with lesbian patients. Their relationships differ in many aspects from heterosexual ones [13]; the interested reader is referred to a review article by MacDonald [14] on the therapeutic implications of such differences. The latter might affect the psychosexual functioning of lesbian couples living with cancer in unique ways.

## The Psychosexual Challenges Faced by Breast Cancer Survivors and their Intimate Partners

In this portion of the article, we have briefly (and not comprehensively, to limit the scope of this work) summarized some of the literature on women’s main biopsychosexual issues that are typically impacted by breast cancer. Both the patient and her intimate partner are faced with dealing with the consequences of cancer and its treatment on the woman’s body, psychological functioning, and sexuality; this is not an easy task. As discussed in the later sections of this article, we are suggesting that this task would be best addressed utilizing a couples group therapeutic modality.

### Psychosexual dysfunction related to breast cancer treatment

A large proportion of cancer patients experiences significant sexual morbidity [15,16]. Sexual problems may worsen with time, especially among those with considerable fear about their illness [17]. Even in a sample of breast cancer survivors considered to be at low risk for psychosocial distress in the year after diagnosis, half of them continued to have sexual dysfunction [18]. Researchers investigating the sexual side effects of medications used to treat cancer have achieved somewhat mixed results. For example, certain drugs (e.g., tamoxifen) have been associated with an increase in vaginal lubrication in some women, as well as with vaginal dryness and decreased libido in others. For those women, such sexual difficulties could be attributed to a possible long-term effect of estrogen deficiency resulting from older age or the disease (as discussed by Kaplan [19]).

### Pain and sexual dysfunction

Pain often develops as a result of cancer and its treatment, and is likely to affect psychosexual health adversely. Newman, Brennan, and Passik [20] found that breast cancer patients experiencing pain due to lymphedema of the ipsilateral arm, a complication of breast cancer treatment, reported decreased sexual drive. The co-occurrence of pain, depression, and fatigue symptoms is common among cancer survivors (Thornton et al. [21]; in turn, this could affect sexual functioning in an adverse manner. Research findings on primary breast cancer survivors’ possible symptoms such as chronic pain, depression, anxiety and chemotherapy-induced premature menopause [22-24] suggest that all these variables are positively related to patients’ sexual problems [25].

### Body image

Researchers targeting the psychosocial impact of breast cancer on sexual functioning have reported the presence of body image problems (which are likely to add to clients’ psychological distress) in this patient population (e.g., Helms et al. [26]). Mastectomy often affects body- and self-image negatively [27]; many women are convinced that it will lead to substantial decrease in sexual desirability. Their fear of revulsion or rejection motivates some of them to avoid showing their scar to their partner, to be extremely sensitive about their partner’s initial response to their body after the surgery, or to stop engaging in sex at all [1]. This reaction could indeed fuel the development of sexual and relationship problems. Understandably, physiological and physical changes affecting areas of the body associated with feminine identity, such as the reproductive system and the breast, could affect women’s sense of femininity adversely. When counseling breast cancer patients, clinicians need to be familiar with these medical issues and should systematically assess whether patients are experiencing psychosexual problems related to the effects of medical treatment for cancer.

### Psychotropic medications

Many patients use psychotropic medications to treat their emotional distress, which is often experienced after receiving a diagnosis and can last for years [28]. The findings of a study conducted by Cullivan et al. [29] on the use of psychotropic medications by cancer patients revealed that about 79 percent of those referred to a psycho-oncology service used such drugs. These medications are often utilized to control several symptoms associated with cancer and its treatment, including depression, pain, nausea, and sleep disturbance [30]. Unfortunately, although some psychotropic medications have a low incidence of sexual side effects [31], many of them are responsible for the development of sexual dysfunction following their use [32,33]. It is necessary to keep in mind that the sexual difficulties of women with breast cancer might be caused or exacerbated by the medications used to treat physical pain and emotional suffering. The next section addresses the cancer-related relationship challenges frequently faced by these couples.

### Intimate relationships and partner response

Breast cancer survivors, if single, often experience difficulties dating [34]. Unattached women are particularly vulnerable, as they might experience isolation and loneliness in addition to their existing problems, especially if they decide to cope with their illness and its consequences by avoiding social and sexual contacts [1]. For patients in a committed relationship, the quality of the communication with their spouses is an important issue. Schag et al. [18] found that women at high risk for psychosocial morbidity following breast cancer treatment are often faced with long-term communication problems with intimate partners. Manne et al. [35] reported that, in couples dealing with cancer, mutual constructive communication is related to higher levels of relationship satisfaction and lower levels of distress for both members. Research conducted in this area by Baum et al. [1] suggests that if the couple is involved in a stable and happy relationship, breast cancer diagnosis and treatment can strengthen the couple’s bond. Clinicians need to consider the importance of providing assistance to oncology couples, not just cancer patients, in handling the adjustment problems related to the cancer [36]. The therapeutic results of focusing on couples could be very positive, in view of the fact that treatment modalities such as couple-focused communication skills training targeting cancer patients and their partners can increase patients’ relationship satisfaction significantly [9].

How do the intimate partners of breast cancer survivors cope? Their psychological reactions are integral to the psychosexual functioning of the couple. There is a significant relationship between spousal and patient adjustment levels, which supports the common notion that partners have a great deal of mutual influence on each other [37]. According to Keller et al. [38], distress levels in cancer patients and spouses are about equally high. Indeed, patients and their spouses do not seem to differ on either levels of symptom distress or hopelessness [39]. Unfortunately, mood disturbance and psychological impairment are experienced by 20 to 30 percent of the spouses of cancer patients [40]: these prevalence rates are similar to those concerning spouses of other seriously medically ill individuals, such as dialysis patients, as 20 percent of these spouses report significant depressive symptoms [41].

The intimate partners of breast cancer survivors are likely to experience emotional distress based on many factors. Their awareness of limitations in how much they can really meet their spouses’ needs is likely to negatively affect them [42]. They also need to deal with their sense of guilt about expressing their feelings and their fears of upsetting or hurting the cancer patient if they were to initiate intimate contact [1]. Spouses are likely to long for encouragement and reassurance by cancer patients that it is acceptable for them to tactfully initiate intimate interactions. In therapy, they should be provided with effective coping skills to deal with possible sexual rejection, and at the same time patients should be instructed on how to communicate that the rejection is likely to be temporary and related to the effects of cancer and its treatment.

Psychosexual challenges are a circular couple phenomenon, as the patient’s intimate partner may interpret the illness-related sexual problems experienced by his mate as sexual rejection. This could initiate a cycle of decreasing sexual contact for the couple, possibly leading to the development of erectile difficulties (as discussed by Goldstein and Teng [43]). To prevent or at least interrupt this vicious cycle, it makes sense to design group intervention strategies targeting the psychosexual needs of couples with breast cancer, as outlined in the rest of the article. It should be noted that, based on our experience in working with these couples in groups, the task of achieving success in couples group therapy with women living with breast cancer could be difficult, especially during the initial psychotherapeutic stages. The aforementioned authors noticed that the effort failed because the women had a very difficult time allowing their husbands to enjoy the focus of the therapist and of the group. Typically, the cancer patients wanted the focus for themselves as they had enjoyed in their own support group. Also, the therapists felt that knowing the women in a different way (i.e., as professionals providing them with full attention) was a major obstacle in couples group therapy. It appeared particularly challenging for cancer patients to tolerate talking about problems already addressed in their prior psychotherapeutic work that they might have to reconsider in this new setting. These difficult clinical experiences highlight the level of complexity of this kind of work, even when the group leaders are highly experienced.

## Couples Group Psychotherapy: A Viable approach to Treating Couples with Breast Cancer

Many of the issues raised in the literature reviewed above have been successfully addressed in a group therapy format throughout the years. Some of the studies published in this area describe effective group interventions targeting body dysmorphic disorder [44], body image problems [45,46] and pain [47,48]. Particularly relevant to the present discussion, the Supportive-Expressive model has been applied successfully to reduce mood and sexual problems of gynecological cancer survivors [49]. However, the aforementioned therapy groups were conducted without inclusion of spouses. In the next paragraphs, we investigate the feasibility of group therapy for couples living with cancer and propose to extend the use of the Supportive-Expressive group therapy model in order to target the specific psychosexual challenges of this patient population.

### Couples groups

Couples group therapy provides an effective way to help couples in distress [50]. It was originally designed in the 1960s to enhance the development of individual identity, reduce the level of couple conflict, and clarify partners’ roles [51]. Within a couples group, patients can observe a variety of coping styles as well as alternatives to dysfunctional behaviors [52]. Couples group therapy from different theoretical perspectives has been found advantageous in dealing with various couples’ challenges, including alcoholism [53], spousal abuse [54,55], post-partum depression [56] and infertility [57].

A couple’s group approach has been found to be a cost-effective way to increase couples’ intimacy and address issues of couple sexuality [58]. It has been successfully applied to treat complex psychosexual challenges such as compulsive sexual behaviors [59,60]. Relative to the treatment of hypoactive sexual desire, couple sex therapy in a group format can ameliorate women’s symptomatology [61]. Additionally, Hurlbert et al. [62] found that a couples-only group was more effective than a women-only group in the treatment of hypoactive sexual desire. Based on the success of couples groups in dealing with many dyadic problems, it makes sense to design interventions that involve oncology patients and their significant others in couples group psychotherapy.

### Group psychotherapy targeting the psychosexual concerns of couples with cancer

Is group therapy with couples a viable treatment modality for addressing the psychosexual needs of breast cancer survivors and their spouses? Within contemporary psycho-oncology, group psychotherapy is a frequently utilized type of intervention[63]; its efficacy as a change agent has been ascertained through extensive empirical research [64]. Research shows that current or past membership in a group is related to greater satisfaction with communication by patients’ husbands and to more communication by the breast cancer survivors [65]. Involvement in psychotherapeutic couples groups should be considered in addition to, or even instead of receiving individual and/or couple therapy. According to a meta-analytic study conducted by Mc Roberts et al. [66] to compare the efficacy of individual and group psychotherapy, group and individual intervention formats yield no significant differences in outcome. The aforementioned authors suggested the utilization of group therapy as a satisfactory cost-effective alternative to individual psychotherapy.

Group psychosocial interventions for cancer patients provide a good setting for the exploration of emotion-laden problems, including those involving sexuality and related difficulties with self-concept, affect management, life goals and relationships [67]. Impairment in sexual relationships is one of the group themes that emerged in oncology psychotherapeutic practice utilizing couples group therapy[68] and should be addressed systematically. To our knowledge, no treatment outcome studies have been conducted to target the specific psychosexual needs of couples living with breast cancer within a couples group setting. To offer a theoretical basis for this research, in the rest of the article we have 1) provided an overview of the manualized, empirically validated Supportive-Expressive model of psychosocial intervention with cancer patients [6,7] and 2) outlined preliminary treatment recommendations extending the use of this model to the treatment of the psychosexual challenges experienced by oncology patients in a couples group format.

### Fundamentals of our supportive-expressive intervention

The main goal of Supportive-Expressive groups is to provide patients with a safe environment for the expression of their deepest concerns, focusing on the here-and-now [69-72]. SEGT is based on the notion that supportive and expressive techniques, when skillfully combined, can shed light on aspects of patients’ lives that are not easily available through introspection [73]. Briefly, this intervention is a semi-structured group therapy program that involves meetings for 90 minutes once a week, led by two co-therapists. Interventions for early stage cancer patients have been structured to last for 12 weeks, while those for people with more advanced disease have run for years as open groups, with new members being added as others leave or die. Therapists are trained to encourage discussion of the major themes listed below, but to avoid didactic presentations. There is no set order or plan to discuss certain themes on certain days, with initial introductions and explanations provided early in the group history, and discussions of termination issues offered in preparation for conclusion. Otherwise, the focus is on conducting a here-and-now interpersonal group directed at social, emotional, existential, interpersonal, and symptom management issues. The SEGT model consists of seven basic components: 1) social support, 2) emotional expression, 3) detoxifying dying, 4) reordering life priorities, 5) family support,6) communication with physicians, and 7) symptom control [74-76].

Leaders of Supportive-Expressive groups benefit from receiving training that aims at enhancing their understanding of the model. This training consists of reading a treatment manual, viewing explanatory videotapes, and participating in a workshop [77]. Findings of a study on these training programs show that the latter are indeed effective, as trained therapists perform better on evaluative tests after each phase of training [78]. In line with the classic guidelines of Gottlieb and Pattison [51], we recommend the use of a co-therapist to avoid possible countertransference problems that may arise due to the delicate and intimate nature of the topics in question. Additionally, co-therapists can provide a healthy model of partner relationship that could be emulated by the couples. Extensive clinical practice with our model by interested therapists is recommended, as this allows them to become increasingly comfortable with cancer-related discussion topics, as well as skilled at making concise statements providing emotional support and inquiring about underlying patients’ emotions.

Researchers have extensively tested our Supportive-Expressive intervention model on women with breast cancer, men and women with HIV infection, as well as families of cancer patients [76]. This model has also been successfully implemented in Europe (e.g., by Reuter et al. [79]). Groups utilizing our model have been running both on a short-term and long-term basis, lasting from three months to several years [6,7] [80,81]. Implementing our model involves using periodically updated treatment manuals [82-84]. Concerning the outcome of SEGT, briefly, this approach has been shown to reduce cancer patients’ psychological distress [77] [85,86], improve coping responses [7], and reduce pain [87,88].Using SEGT has also resulted in longer survival time among women with metastatic breast cancer [81] especially those with estrogen-receptor negative disease[89].The effect of psychosocial treatment on survival time has been demonstrated in half of the published randomized trials [90]. In comparing SEGT with cognitive-behavioral group treatment of HIV-infected individuals, Kelly et al. [91] found the Supportive-Expressive intervention to be more effective.

No formal randomized trials exist on specific application of this model to cancer couples living with psychosexual dysfunction. However, we have run both spouse and couples’ groups over the past two decades, and found them to be well-accepted and helpful to our patients in addressing existential concerns, managing overwhelming affect, and improving family relationships. Thus, we are optimistic that the application of our model to the various psychosexual challenges faced by couples with cancer could lead to positive outcomes. Because SEGT is an example of an intervention that is multidimensional and multidisciplinary, we encourage interested physicians, nurses, counselors, social workers, and various health care providers to implement the following suggestions on how to help couples with breast cancer address the multiple psychosexual challenges that they often encounter.

In the following paragraphs, we briefly describe how the seven components of the model can be linked to the psychosexual issues raised in the literature review conducted above. We also present several change strategies to be used when running couples therapy groups, relating our treatment recommendations to the model’s components. Some of our suggestions are only indirectly related to the sexual functioning of couples with cancer, as they target primarily their psychosocial challenges. Nonetheless, once illness-related psychopathology and social isolation decrease, issues of sexual functioning may surface, and can be addressed through the adoption of a couple’s group therapy format.

#### Social support

Social support that is truly meaningful provides a crucial link between disease outcome and medical care [76], as it may influence cancer progression positively via changes in the cellular immune response, both at the tumor level and in peripheral blood [92]. On the other hand, Koopman et al. [93] found that aversive support represents another source of life stress related to cancer patients’ emotional distress. Furthermore, social support (measured in the previous study by the size of patients’ support system) is significantly related to lower mood disturbance for patients who report greater previous life stress than their counterpart. These findings underscore the fact that breast cancer is often experienced as a very traumatic event, particularly for patients with unsupportive social environments and numerous past life stressors [94]. We believe that the examination of intimate difficulties in a couples group format provides a very important opportunity to ‘normalize’ them [72]. By exposing oncology couples to the intimate (including the sexual) challenges faced by other couples living with cancer, dyadic problems are framed in a broader context rather than just in the interpersonal history of one relationship. Once exposed to others voicing similar sexual difficulties, couples or individual partners who are at first reluctant to address their sexual problems might become comfortable discussing their sexual issues in an accepting atmosphere. In turn, they might find that the support received benefits their sexual lives; research is certainly needed in this area.

For both breast cancer survivors and their intimate partners, levels of social support from family and friends are significantly related to psychosocial adjustment [95]. Psychotherapy, especially in groups, can provide a new social network in which participants share a common bond [74]. Group psychotherapeutic support provides a new and important social connection at a time when illness makes a person feel removed from the flow of life and many others withdraw due to awkwardness or fear. Indeed, the very thing that damages other social relationships affords admission to such groups, providing a surprising intensity of caring among members right from the beginning. Thus, constructing new social networks for cancer patients and their families via support groups and other means is doubly important: it occurs during a timewhen natural social support may erode, and when additional support is needed [96]. Participation in a psychotherapeutic couples group becomes an important social connection, helping couples avoid retreating out of fear of death.

The benefits of social support within a group therapy format can also affect the self-concept of cancer survivors and their spouses. Cancer and its treatment are likely to challenge self-concept; if people join a therapy group, they can improve the way they view themselves by relying on group membership [97]. Given the many challenges faced by couples with cancer, the use of a collective identity for the enhancement of self-concept may prove to be an important protective factor. An additional way in which Supportive-Expressive groups encourage mutual support is by promoting group members’ socialization outside therapy sessions and through personal visits [6,76].

Couples group discussion should also focus on ways in which spousal distress could be minimized through enhancing their social support system. In this regard, a possible function served by patients’ spouses is to assume a supportive role to other spouses who are coping with cancer-related challenges. The restriction of activities due to patients’ cancer as well as spouses’ mood disturbances are understandably related to increased spousal negative behaviors [98]. Therefore, it makes sense to emphasize the benefits of spouses getting together to socialize, in order to reduce their common social isolation that, in turn, could lead to the development of relationship problems. Once spouses become comfortable with other spouses, they could support each other in helpful confidential discussions of psychosexual problems that perhaps they would not share in a therapeutic setting.

#### Emotional expression

Unfortunately, the expression of emotion is often an aspect of cancer patient adjustment that is overlooked or suppressed. Emotional suppression and avoidance are associated with poorer coping among cancer patients [99-101]. Emotional suppression also reduces intimacy in families and limits opportunities for direct expression of affection and concern. At the same time, there is much that can be done in both group and individual psychotherapies to facilitate the expression of emotion appropriate to the disease. Doing so seems to reduce the repressive coping strategy that lessens expression of positive as well as negative emotion. Emotional distress in patients with a serious medical condition could lessen once these individuals are provided with a safe forum facilitating greater openness through the exploration and expression of feelings and thoughts [69]. This process could include disclosingpainful feelings related, for instance, to patients’ low sexual desire after treatment and to expression of emotions related to sexual problems. When handled delicately by an experiencedcouples group therapist, thiswork could lead to the amelioration of such problems; research is needed in this area. Among women with advanced breast cancer, emotional expressiveness is associated with better psychological adjustment [102], as those patients who are able to ventilate strong feelings directly cope better with cancer [75,100,103]. There is also evidence from a randomized clinical trial that participation in SEGT results in reduced suppression of emotional expression [104], and that this reduced expression of emotion mediates the beneficial effect of SEGT in reducing distress [85].

The use of the psychotherapeutic setting to deal with painful affect also provides an organizing context for handling its intrusion. When intrusive thoughts involving fears of death and dying occur, they can understandably create a barrier to sexual intimacy, and could be better managed by patients and families who know that there is a time and a place during which such feelings will be expressed and dealt with. Furthermore, disease-related dysphoria may become even more intense when amplified by isolation, leaving patients to feel that they are deservedly alone with the sense of anxiety, loss and fear that they experience. Being in a group where many others express similar distress normalizes their reactions, making them less alien and overwhelming.

Outcome research on the utilization of the model with breast cancer patients indicates that participation in a 12-week psychotherapeutic group with sessions lasting 90 minutes is significantly related to a decrease in total mood disturbance, anxiety, depression, and impact of cancer on patients’ lives [77]. These findings suggest that fostering emotional expression, a primary goal of this group therapy approach, is crucial for the reduction of patients’ psychopathology, thus potentially affecting their sexuality in a positive way. Furthermore, research evidence shows that, during times in which patients feel that their health is improving, group discussions become more centered around issues such as patients’ self-image [105]. By being able to verbalize personal concerns and feelings related to altered body image, which could become problematic as medical treatment often alters patients’ bodies, cancer survivors can achieve a successful integration of a changed body and self-image [69]. Patients’ sensitivity about their partners’ response to the effects of surgery, their feelings decreased sexual desirability, and the corresponding avoidance of sexual interaction identified in the literature [1] are all likely to decrease once issues of body image are successfully addressed.

A group environment can provide a ‘relationship laboratory’ for experimenting with risk-taking involving disclosure and feedback about emotional issues that could then be applied to family and other relationships [72]. Emotional expression helps group participants test responses from others and manage their strong feelings. For example, patients’ intimate partners (or patients themselves) may try to cope with multiple cancer-related stressors by self-medicating through heavy alcohol use. For these people, it is particularly important to become involved in some form of group therapy, as alcoholism is one of the challenges that could be successfully addressed through implementation of couples group interventions [53] and can affect sexual health negatively [106].

In case the clinician decides to run couples groups in which asymptomatic women as well as women with terminal cancer are present, a group member could eventually be in critical condition. Under these circumstances, it is particularly important for therapists to gently encourage individuals at all stages of the cancer to assist each other in coping with the patient’s impending death. This process could also have a positive impact on patients’ spouses and their families [6]. Interestingly, when intimate partners report lower mood disturbance, cancer patients do the same [107]. This finding highlights the need to provide spouses with a way to successfully deal with their own distress, also for the sake of patients’ emotional wellbeing. As many spouses experience guilt about expressing their feelings and fear of hurting or upsetting their ill wives if they were to initiate sexual contact [1], an open group discussion of such feelings is likely to lead to resolution of some of these delicate issues.

#### Detoxifying dying

The concerns of most patients who are seen in conventional group therapy settings are likely to differ from those of cancer patients, due to the shared intimacy with mortality experienced by the latter [76,108]. Unfortunately, physical problems associated with serious medical conditions have various psychological implications, including significant distress about a foreshortened future [109]. Facing life-threatening issues directly, in a Supportive-Expressive therapeutic setting, group members can detoxify fears of dying by discussing their anxiety about death, shifting from emotion-focused to problem-focused coping [76,110]. By the same token, intimate partners’ anxieties associated with the possibility of their spouses’ death could be addressed in-depth during couple’s group therapy. In this type of therapeutic setting, it is important to discuss and demystify spouses’ fear that sexual activity could do harm and even possibly shorten their wives’ lives. Literature in support of “detoxifying death” within couple’s group interventions for enhancing sexuality is lacking. However, it is possible that cultivating commitment and openness among these couples within a caring couplesgroup therapy setting could help themface their mortality. At the same time, it could facilitate the development of an even stronger emotional bond that could have positive repercussions on the couples’ sex lives. Interested scholars are encouraged to investigate this highly neglected research area, which combines two common taboos, sex and death.

Death anxiety in particular is intensified by isolation, in part because we often conceptualize death in terms of separation from loved ones. Feeling alone, especially at a time of strong emotion, makes one feel already somewhat dead, setting off a cycle of further anxiety. These feelings could be transformed by psychotherapeutic techniques that directly address such concerns.This component of SEGT involves examining the threat of death directly rather than avoiding it, which often yields a new perspective [109]. When worked through, life-threatening problems seem lessoverwhelming [76,80], as the process of dying is often more threatening than death itself. Direct discussion of death anxiety could help divide the fear of death into a series of problems, including loss of control over treatment decisions, fear of separation from loved ones, and anxiety about pain. Discussion of these concerns can lead to a means of addressing, if not completely resolving, each of these issues. Thus, facing death could result in positive life changes. Even the process of grieving can be simultaneously reassuring and threatening. The experience of grieving others who have died of the same condition constitutes a deeply personal experience of the depth of loss that will be experienced by others after one’s own death. Similarly, spouses face their potential losses by watching others grieve, while learning that such losses can be worked through [80,110-111].

Supportive-expressive groups can perform several functions, including placing death into perspective and demystifying the process of dying [6,70]. As already mentioned, group members often choose to pay personal visits to patients, including when the latter are at the final stage of cancer. Such visits could reveal very graphic and intense details about the last days of a patient’s life. As group members provide dying patients and their families with social support by visiting with them or by keeping in close telephone contact with their family members/caregivers, this offers them an opportunity to experience death vicariously. Many group members perceive such a circumstance as very valuable, possibly due to the fact that it may provide a sense of being more in control over the dying process.

As a result of feelings of safety and freedom within the group, couples could share stories related to the death of former group members, including descriptions of the specific type of help these women received from their spouses. For example, during one session with metastatic breast cancer patients, some patients provided detailed information on ways in which the husband of a patient who had just died had assisted her through the final stage of her illness (as not all group members had paid personal visits to the couple). The descriptions included very graphic details, such as how he had helped the patient position her body and hold the position in such a way that the blood she was regurgitating at times (due to her terminal condition)did not suffocate her. The description of this man’s enormous care, patience, and devotion to his wife was a very emotional experience for all the people present at that meeting. It included vivid details on creative and loving ways in which he remained by his wife’s side in a supportive role until the end, regardless of the gravity of the situation.

The tendency for SEGT patients to focus on death-related details has been investigated in the literature. Specifically, as some patients’ health deteriorates, the topics brought up by group members fall in the categories of death and dying and medical treatment, as opposed to them engaging in small talk, as well as in discussion of family situations and self-image issues, which usually occurs when patients’ health is improving [105]. Following a diagnosis of cancer, a variety of coping strategies emerge, including positive reappraisal and cognitive avoidance [112]. Denial and avoidance have their consequences, including an increase in anxiety and isolation. It is likely that the use of avoidance and denial becomes less feasible when the deterioration of the health of a group member reaches its final stage; this situation focuses the attention of the group on more relevant, cancer-related issues. Excessive use of defenses such as avoidance and denial in the face of the death of a group member would also prevent patients’ intimate partners from identifying practical ways in which they can assist their spouses, as a resultof group discussion on this topic.

Perhaps the provision of graphic, death-related descriptions could help group members feel somewhat in control of a situation that is hardly ever under control. The fact that a husband can be present and attentive in providing support to his dying wife may instill precious hope in couples. Moreover, it could provide a modeling experience to patients’ spouses, who often doubt their ability to provide adequate support to their wives as the health of the latter deteriorates. Despite the fact that discussions like the one described above are hardly related to sexual intimacy on a superficial level, sharing such intense experiences could represent a powerful bonding tool that is likely to enhance couples’ emotional closeness. This, in turn, could affect closeness on all levels, including sexually, reminding both partners of the importance of focusing together on the here-and-now in pleasurable ways.

#### Reordering life priorities

Closely related to the prior component, reordering life priorities within a couples group format involves focusing on what medically ill patients and their spouses can actually do to prepare for the possibility of patient’s death, as its acceptance carries with it an opportunity for re-evaluating life priorities. When a cure is not possible, a realistic evaluation of the future could help those with life-threatening illness make the best use of the remaining time. Reordering priorities should also include making sexual fulfillment, to the extent possible, a shared goal within a couple living with the consequences of cancer. One of the costs of unrealistic optimism is the loss of time for accomplishing life projects, communicating openly with family and friends, and setting affairs in order. Facing the threat of death can aid in making the most of life [75-76,108]. This could help patients take control of those aspects of their lives that they can influence, while grieving and relinquishing those they cannot; establishing a domain of control could be quite reassuring.

It is very important for cancer survivors to use the rest of their precious time well [69], and for their intimate partners to be able to do so without being overpowered by the thought of their spouses’ possible death. A group therapy format provides individuals with the opportunity to engage in extensive discussions focused on clarifying the meaning of life on a personal basis and on maximizing the quality of the remaining life span [6]. A limited sense of future could inspire couples to reconsider how they are living their lives and to discuss whether they want to modify their priorities. Several Supportive-Expressive group members throughout the years have made significant life changes, including leaving their jobs and dedicating more time to their loved ones (which can include intimate/sexual activities), as well as returning to school and becoming experts in areas that always attracted them, such as the graphic arts. By achieving these goals, patients can show to other group members that it is indeed possible to decide to spend the rest of one’s life focusing on something interesting and completely new in spite of the illness. This renewed life focus could allow patients to feel a sense of accomplishment that they might have never experienced, had they not been faced with a serious medical condition.

Overall, the possibility of death could motivate patients to focus on some important yet previously underestimated issues, as well as on new pursuits that can enhance the rest of their lives. This task can become even more enjoyable if patients’ spouses contribute to the achievement of these new goals. Many intimate partners feel unprepared and overwhelmed by the cancer and its consequences on their family lives; the shared vision of new life achievements could counteract such feelings of helplessness. Furthermore, spouses are often negatively affected by a sense of powerlessness in terms of being able to assist their spouses during illness[42] and by having to engage in multiple new tasks related to helping the patient manage new challenging situations [40]. To address this issue, it may be beneficial for patients’ spouses to discuss during group sessions how to reorder their own life priorities. This could include, for example, how to prioritize specific domestic tasks (depending on one’s family and household needs), as well as romantic activities leading to fulfilling sexual interactions. Patients could help their spouses achieve these goals, and working together on projects may in turn bond couples more closely and affect the quality of their romantic and sexual interactions in positive ways.

#### Family support

Addressing common patients’ dilemmas in a couples group format, such as problems in communicating clearly with family members, could enhance family support for cancer patients and their loved ones. According to Giese-Davis and colleagues[107], when cancer patients rate their intimate relationship as high in cohesion-expression and in conflict, they also report lower levels of distress (which, in turn, are related to better sexual functioning even after controlling for psychological symptoms and relationship quality[113]). As a possible implication of these findings, Giese-Davis and colleagues proposed that women with cancer might need to address conflicts and difficulties with their intimate partners in an open manner in order to lower their own distress level. Additionally, they indicated that, to achieve such a goal, treatment may have to focus more on the couple’s relationship and level of coping than on patients’ individual coping. This is in line with what we propose in the present article: to provide psychosexual group treatment to patients living with a serious medical condition, such as breast cancer, by addressing these delicate issuesin couples groups. Addressing in a couples group setting how, for instance, a husband copes with his erectile problems stemming from his fear of hurting his wife via sexual interaction and how his wife copes with his sexual difficulties, could facilitate the resolution of such sexual challenges; research is certainly needed in this area.

Directly related to the emotional expression component of the Supportive-Expressive model, psychotherapeutic interventions should focus on ways in which to improve the quality of the communication between cancer patients and their intimate partners. Supportive-Expressive groups can be quite helpful in this regard; in turn, enhanced communication skills may elicit better verbalization of needs and higher adjustment to new medical, social, vocational, and financial realities. An atmosphere of open and shared problem-solving in families results in reduced anxiety and/or depression among cancer patients [10,107,114].Thus, facilitating the development of such openness is a useful therapeutic goal. Our group format is especially helpful for this task, in that difficulties expressing needs and wishes could be examined among group members as a model for clarifying communication in the family.

Once the relationship is happier and more stable, couples could feel even closer than they were prior to the experience of cancer [1]. The fact that breast cancer patients often find it hard to share their feelings and thoughts with their spouses [115,116] is a challenge that should become a priority in treatment. In turn, once psychological distress is minimized, patients are less likely to need to use psychotropic medications, which are often responsible for the development of sexual dysfunction [32,33]. Supportive-Expressive couple’s group therapy addresses all these issues by providing a safe forum in which both patients and spouses could feel free to bring up delicate issues related to the aforementioned topics.

Couples struggling with communication and marital difficulties can receive valuable feedback from other couples who have found creative ways to resolve similar challenges. In this regard, therapists could help couples deal with these issues successfully by providing communication skills training, modeling high-quality interaction strategies, and encouraging group members to develop innovative solutions to potential communication dilemmas. Additionally, couples groups can also function as a model to be used to clarify communication problems within the patients’ family beyond the dyadic relationship. When a group member asks for feedback on how to deal more effectively with another family member, individuals of both genders could express an alternative point of view that could arouse more defensiveness if it came from the patient’s family member in question. The suggestions provided by the group could them be used outside the family environment to address family conflicts in more productive ways.

Participating in a group also helps patients and their partners develop “role flexibility”, i.e., the capacity to exchange roles, or to develop new ones, based on the demands of the illness. For example, a breast cancer survivor shared in one of the Supportive-Expressive group sessions that she had written an “owner’s manual” about their home for her husband, so that he could be more effective with household chores. These new activities could provide spouses with a sense of having an important role in patients’ recovery process. Furthermore, the strategy of creating manuals to address complex domestic issues, when implemented by couples living with cancer, could free up time for patients and their spouses, allowing them to engage in romantic interactions more often.

#### Communication with physician

Difficulties with sexual functioning may begin as problems with the disease, such as fatigue and pain, and with its treatment, e.g., vaginal dryness secondary to estrogen blockage. The last two components of the SEGT model, i.e., communication with physicians and symptom control, could be utilized to address these issues, as our model stresses the importance of providing comprehensive, multilevel and multidisciplinary care to cancer patients and their spouses. It is essential to find effective ways to facilitate cancer patient-medical health provider communication (as discussed by Caldwell et al. [117]). To this end, supportive-Expressive groups can be quite useful, providing mutual encouragement to: get questions answered; participate actively in treatment decisions; and consider alternatives carefully. Research indicates that patients who do so are less distressed over the long term, regardless of their treatment decisions (e.g., lumpectomy and radiation versus mastectomy[118]). Such groups must be careful not to interfere with medical treatment and decisions, but rather to encourage clarification and the development of a cooperative relationship between doctor and patient [119].

Group participation could improve the quality of communication of both the patient and her partner with their health care professionals. This could be achieved in group sessions by clarifying both the couple’s goals and the means of expressing them when dealing with health providers. Couples are encouraged to discuss many delicate topics with their physicians/health care providers, including fertility problems stemming from chemotherapy treatment and the use of psychotropic medications. Such group discussions may also include considering the utilization of psychotropic medications by the spouse, at least temporarily, in order reducing partner’s distress level. However, it should be noted that cancer-related medical treatment and psychotropic medication use, as discussed earlier, can both result in the development of sexual dysfunction. Couples should decide in collaboration with their medical providers whether it would be appropriate to modify their medication regimen in order to minimize potential sexual side effects for both partners. Again, prior to addressing these sensitive issues in a doctor’s office, it is important for couples to have a good understanding of the feelings, needs, and concerns underlying these topics. Acouples group forum could be very helpful in this regard. Patients’ intimate partners are likely to benefit from learning how to deal effectively with medical staff and to contribute to important decisions concerning cancer management, as they often long for ways to support their spouses in constructive manners.

#### Symptom control

Numerous physical symptoms, including chronic pain and chemotherapy-induced premature menopause, are significantly related to sexual dysfunction among breast cancer patients (as discussed by Laganá et al. [25]). There are various symptom control techniques that could be utilized as therapeutic change strategies within a couples group format, including self-hypnosis, progressive muscle relaxation, guided imagery, biofeedback, and meditation. These tools provide medically ill patients and their significant others with coping skills that can enable them to better manage both physical cancer-related symptoms and psychological symptoms, such as anxiety [110]. When weekly SEGT includes cognitive techniques such as self-hypnosis training to manage symptoms, cancer patients report lower levels of self-rated pain sensation and suffering than group members not exposed to the training [88]. Such cognitive techniques focus on methods to identify emotions as they develop, analyze sources of emotional response, and move from emotion-focused to problem-focused coping. These approaches help patients take a more active stance toward the illness, as they learn to divide seemingly insurmountable problems into smaller and more manageable ones. Given the high stress level often experienced by patients’ spouses (as well as high depressive symptomatology, as reported by Braun et al.[120]), their utilization of these coping strategies could be beneficial in reducing their symptomatology; research is needed on this topic.

A description by group members of their successful interactions with health professionals regarding how to best control their symptoms could provide positive modeling experiences to couples. A cooperative partnership among these professionals, the patient, and her spouse is necessary, especially if the couple wishes to address sexuality concerns related to inadequate symptom control such as unmanaged sexual side effects of cancer medical treatment. For example, couples could discuss with their sexual health care provider the development of symptoms of dyspareunia following chemotherapy, as well as ways in which this sexual side effect could be bypassed to the point that it no longer interferes with sexual functioning. We encourage the professionals in question to instruct couples to experiment with alternative ways in which to interact intimately, in order for them not to rely necessarily on intercourse and vaginal stimulation for sexual pleasure, at least until vaginal lubrication difficulties related to medical treatment are ameliorated (as atrophic vaginitis is a challenging problem for many patients). Only then will it be appropriate to address the possible aversive conditioning of the cancer survivors’ sexual response that may exacerbate sexual difficulties such as dyspareunia.

Overall, SEGT aims at facilitating patients’ enhancement of their quality of life [69]; the latter is likely to improve if sexual issues are no longer problematic. The sexual symptoms of cancer survivors should be thoroughly addressed in couples groups. Targeting patients’ sexual difficulties (such as low sexual desire) within a couples group format appears to be more successful than providing women-only group therapy [62]. Anticipatory nausea and pain are likely deterrents to intimate interaction with a partner, especially when these symptoms are added to vaginal lubrication difficulties, often precipitated by cancer medical treatment. If couples receive psychotherapeutic assistance in managing these complex side effects, the likelihood of their sexual dysfunction and related marital problems may be reduced. The symptom control techniques mentioned above might facilitate the engagement in sexual activities by the cancer survivor, once her symptomatology is under control enough for her to desire to do so. In turn, the maintenance of a stable frequency of sexual interaction, again not limited to intercourse, is likely to strengthen the bond between the patient and her intimate partner. This could prevent or, at least, minimize the development of relationship difficulties that might follow a drastic reduction in sexual activity.

## Conclusions

The results of empirical research show that group psychotherapy is a successful treatment modality [64], and it is often used within oncology psychotherapeutic practice [63]. Couple-based coping training interventions with early-stage breast or gynecological cancer survivors and their partners aimed at facilitating their psychosexual adjustment appear to be more effective than individual training [121]. It is possible that such a finding could be extended to couples group interventions like the one proposed herein, but this needs to be tested experimentally in future research. Without this empirical evidence, we are not yet in the position to determine how generalisable all the components of the Supportive Expressive model are to different categories of breast cancer patients and to sexual problems. As already mentioned, this model has been successful at addressing the sexual difficulties of gynecological cancer survivors (as reported by Caldwell et al. [49]), but the treatment occurred in a group format, not in a couples group format. Certainly, clinicians targeting the sexual problems of couples with breast cancer (and other kinds of cancer) are faced by complex challenges, given the often-complex clinical symptomatology of their clients. Indeed, throughout the treatment of their illness, cancer patients frequently undergo painful and stressful medical procedures that typically have psychological, physical, and sexual consequences (as discussed by Laganá et al. [122]).It is important to involve cancer patients’ sexual partners in psychosocial oncology research and treatment efforts, in view of the complex interplay between patients’ and partners’ psychosexual issues [123].

To offer a contribution to couplesgroup therapy work in this challenging clinical area, in the present article we have proposed extending the utilization of a manualized, empirically validated therapy model to address the psychosexual needs of couples with breast cancer within a couples group format. Our clinical recommendations can be viewed as preliminary guidelines to provide psychosexual therapeutic care to couples with a serious medical illness. Through participation in couples group therapy, patients and their intimate partners could learn a) many coping styles to substitute dysfunctional behaviors and practices with more effective and healthy ones[52], as well asb) strategies to enhance their sexual functioning. Although our group psychotherapy model was not originally designed to address couples’ psychosexual challenges, it may provide a helpful tool through which these difficulties can be targeted.

Clinicians treating this patient population need to be skilled at addressing couples’ multiple psychosexual concerns in a direct manner, and they should also provide expert advice on appropriate lovemaking techniques (as pointed out by Schover [124]). In the present article, we have provided some examples of how the SEGT model could be helpful in dealing with such issues. Given the lack of studies on the sexual benefits of couples group SEGT therapy, research is definitely needed to test whether our suggestions would lead to measurable improvements in sexual functioning. Researchers also need to identify medical and psychosocial variables that are associated with helping patients and their partners remain healthy in both their individual sexual functioning and in their sexual balance as a couple. Interestingly, in the aforementioned study by Fobair et al. [4], Latinas reported fewer sexual problems after medical treatment of their cancer than Euro-American patients, contradicting some prior findings (e.g., those of Spencer et al. [125]). The need for research in this area concerning cultural variables, including acculturation, is particularly evident, given the paucity of studies on this topic. Culturally sensitive intervention programs exist in this area, such as the SPIRIT program, which combines a written workbook and peer counseling for African-American breast cancer survivors. Yet, positive clinical outcomes are limited, as sexually active women reported better sexual functioning at 6-month follow-up, but not by year 1 [126]. Based on theoretical models such as the SEGT model discussed herein, methodologically sound psychotherapeutic efforts should include the development of multidisciplinary interventions aimed at counteracting the adverse psychosexual impact of the disease. Indeed, the most effective type of intervention for the treatment of sexual problems in survivors of breast cancer is, according to a recent review of the literature conducted by Taylor et al. [127], both couple-based and psycho-educational in nature, and includes an element of sex therapy. However, this is not the same as running couples groups for this patient population: if clinicians are up for this challenging group therapy task that has yet to be contrasted empirically with more conventional therapy methods, the results could be rewarding. Hopefully, the suggestions that we have provided in this article will be useful to pioneers of couples group therapy interventions for psychosexual problems.

## References

[1] Baum M, Saunders C, Meredith S (1995). Breast cancer: A guide for every woman.

[2] Saevarsdottir T, Fridriksdottir N, Gunnarsdottir S (2010). Quality of life and symptoms of anxiety and depression of patients receiving cancer chemotherapy: longitudinal study. Cancer Nurs.

[3] Thompson J, Coleman R, Colwell B, Freeman J, Greenfield D (2013). Levels of distress in breast cancer survivors approaching discharge from routine hospital follow-up. Psychooncology.

[4] Fobair P, Stewart SL, Chang S, D’Onofrio C, Banks PJ (2006). Body image and sexual problems in young women with breast cancer. Psychooncology.

[5] Alicikus ZA, Gorken IB, Sen RC, Kentli S, Kinay M (2009). Psychosexual and body image aspects of quality of life in Turkish breast cancer patients: a comparison of breast conserving treatment and mastectomy. Tumori.

[6] Spiegel D, Yalom ID (1978). A support group for dying patients. Int J Group Psychother.

[7] Spiegel D, Bloom JR, Yalom I (1981). Group support for patients with metastatic cancer. A randomized outcome study. Arch Gen Psychiatry.

[8] Abasher SM (2009). Sexual health issues in Sudanese women before and during hormonal treatment for breast cancer. Psychooncology.

[9] Manne SL, Ostroff JS, Winkel G, Fox K, Grana G (2005). Couple-focused group intervention for women with early stage breast cancer. J Consult ClinPsychol.

[10] Giese-Davis J, Collie K, Rancourt KM, Neri E, Kraemer HC (2011). Decrease in depression symptoms is associated with longer survival in patients with metastatic breast cancer: a secondary analysis. J ClinOncol.

[11] Fobair P, Koopman C, DiMiceli S, O’Hanlan K, Butler LD (2002). Psychosocial intervention for lesbians with primary breast cancer. Psychooncology.

[12] Fobair P, O’Hanlan K, Koopman C, Classen C, Dimiceli S (2001). Comparison of lesbian and heterosexual women’s response to newly diagnosed breast cancer. Psycho-Oncology.

[13] Igartua KJ (1998). Therapy with lesbian couples: the issues and the interventions. Can J Psychiatry.

[14] MacDonald BJ (1998). Issues in therapy with gay and lesbian couples. J Sex Marital Ther.

[15] Kornblith AB, Ligibel J (2003). Psychosocial and sexual functioning of survivors of breast cancer. SeminOncol.

[16] White ID, Allan H, Faithfull S (2011). Assessment of treatment-induced female sexual morbidity in oncology: is this a part of routine medical follow-up after radical pelvic radiotherapy?. Br J Cancer.

[17] Urbánek V, Weiss P, Kofránek J, Albl M (1994). [Sexual function in women with breast carcinoma in relation to the time interval after mastectomy]. CeskaGynekol.

[18] Schag CA, Ganz PA, Polinsky ML, Fred C, Hirji K (1993). Characteristics of women at risk for psychosocial distress in the year after breast cancer. J ClinOncol.

[19] Kaplan HS (1992). A neglected issue: the sexual side effects of current treatments for breast cancer. J Sex Marital Ther.

[20] Newman ML, Brennan M, Passik S (1996). Lymphedema complicated by pain and psychological distress: a case with complex treatment needs. J Pain Symptom Manage.

[21] Thornton LM, Andersen BL, Blakely WP (2010). The Pain, Depression, and Fatigue Symptom Cluster in Advanced Breast Cancer: Covariation with the hypothalamic-pituitary-adrenal axis and the sympathetic nervous system. Health Psychol.

[22] Ganz PA, Rowland JH, Desmond K, Meyerowitz BE, Wyatt GE (1998). Life after breast cancer: understanding women’s health-related quality of life and sexual functioning. J ClinOncol.

[23] Ochsenkühn R, Hermelink K, Clayton AH, von Schönfeldt V, Gallwas J (2011). Menopausal status in breast cancer patients with past chemotherapy determines long-term hypoactive sexual desire disorder. J Sex Med.

[24] Schover LR (1994). Sexuality and body image in younger women with breast cancer. J Natl Cancer InstMonogr.

[25] Lagana L, Classen C, Classen N, Koopman C, Spiegel D Chemotherapy-induced premature menopause and chronic pain as predictors of psychological and sexual difficulties among primary breast cancer survivors.

[26] Helms RL, O’Hea EL, Corso M (2008). Body image issues in women with breast cancer. Psychol Health Med.

[27] Fries A, Reinhard G (1996). [Effects of mastectomy on dimensions of psychological and psychosocial experience and behavior of affected women]. Rehabilitation (Stuttg).

[28] Coyne JC, Palmer SC, Shapiro PJ, Thompson R, DeMichele A (2004). Distress, psychiatric morbidity, and prescriptions for psychotropic medication in a breast cancer waiting room sample. Gen Hosp Psychiatry.

[29] Cullivan R, Crown J, Walsh N (1998). The use of psychotropic medication in patients referred to a psycho-oncology service. Psychooncology.

[30] Kim SW, Shin IS, Kim JM, Kim YC, Kim KS (2008). Effectiveness of mirtazapine for nausea and insomnia in cancer patients with depression. Psychiatry ClinNeurosci.

[31] Segraves RT (1998). Antidepressant-induced sexual dysfunction. J Clin Psychiatry.

[32] Hirschfeld RM (1999). Management of sexual side effects of antidepressant therapy. J Clin Psychiatry.

[33] Shen WW, Hsu JH (1995). Female sexual side effects associated with selective serotonin reuptake inhibitors: a descriptive clinical study of 33 patients. Int J Psychiatry Med.

[34] Ganz PA, Coscarelli A, Fred C, Kahn B, Polinsky ML (1996). Breast cancer survivors: psychosocial concerns and quality of life. Breast Cancer Res Treat.

[35] Manne SL, Ostroff JS, Norton TR, Fox K, Goldstein L, Grana G (2006). Cancer-related relationship communication in couples coping with early stage breast cancer. Psychooncology.

[36] Northouse LL, Templin T, Mood D, Oberst M (1998). Couples’ adjustment to breast cancer and benign breast disease: a longitudinal analysis. Psychooncology.

[37] Northouse LL, Dorris G, Charron-Moore C (1995). Factors affecting couples’ adjustment to recurrent breast cancer. SocSci Med.

[38] Keller M, Henrich G, Beutel M, Sellschopp A (1998). [Mutual stress and support in couples with one cancer patient]. PsychotherPsychosom Med Psychol.

[39] Northouse LL, Laten D, Reddy P (1995). Adjustment of women and their husbands to recurrent breast cancer. Res Nurs Health.

[40] Blanchard CG, Albrecht TL, Ruckdeschel JC (1997). The crisis of cancer: psychological impact on family caregivers. Oncology (Williston Park).

[41] Rideout EM, Rodin GM, Littlefield CH (1990). Stress, social support, and symptoms of depression in spouses of the medically ill. Int J Psychiatry Med.

[42] Samms MC (1999). The husband’s untold account of his wife’s breast cancer: a chronologic analysis. OncolNurs Forum.

[43] Goldstein MK, Teng NN (1991). Gynecologic factors in sexual dysfunction of the older woman. ClinGeriatr Med.

[44] Wilhelm S, Otto MW, Lohr B, Deckersbach T (1999). Cognitive behavior group therapy for body dysmorphic disorder: a case series. Behav Res Ther.

[45] Grant JR, Cash TF (1995). Cognitive-behavioral body image therapy: Comparative efficacy of group and modest-contrast treatments. BehavTher.

[46] Lewis VJ, Blair AJ, Booth DA (1992). Outcome of group therapy for body-image emotionality and weight control self-efficacy. BehavPsychother.

[47] Keel PJ, Bodoky C, Gerhard U, Müller W (1998). Comparison of integrated group therapy and group relaxation training for fibromyalgia. Clin J Pain.

[48] Thomas VJ, Dixon AL, Milligan P (1999). Cognitive-behaviour therapy for the management of sickle cell disease pain: An evaluation of a community-based intervention. Br J Health Psychol.

[49] Caldwell R, Classen C, Lagana L, McGarvey E, Baum L (2003). Changes in sexual functioning and mood among women treated for gynecological cancer who receive group therapy: A pilot Study. J ClinPsychol Med S.

[50] Lakoff RS, Baggaley A (1994). Working with couples in a group: Theoretical and practical issues. Group Anal.

[51] Gottlieb A, Pattison EM (1966). Married couples group psychotherapy. Arch Gen Psychiatry.

[52] Feld BG (2003). Phases of couples group therapy: A consideration of therapeutic action. Group.

[53] O’Farrell TJ, O’Farrell TJ (1993). A behavioral marital therapy couples group program for alcoholics and their spouses. Treating alcohol problems: Marital and family interventions.

[54] Brannen SJ, Rubin A (1996). Comparing the effectiveness of gender-specific and couples groups in a court-mandated spouse abuse treatment program. Res Social Work Prac.

[55] Stith SM, Rosen H, McCollum EE, Thomsen CJ (2004). Treating intimate partner violence within intact couple relationships: Outcomes of multi-couple versus individual couple therapy. J Marital FamTher.

[56] Gruen DS (1993). A group psychotherapy approach to postpartum depression. Int J Group Psychother.

[57] Tuschen-Caffier B, Florin I, Krause W, Pook M (1999). Cognitive-behavioral therapy for idiopathic infertile couples. PsychotherPsychosom.

[58] Friedenberg MM (1999). A couples intimacy group, integrating feminist and systemic principles within a structured cognitive behavioral format. Practicum Report for Degree of Master of Social Work.

[59] Risen CB, Althof SE (1990). Couples group psychotherapy: Rebuilding the marital relationship following disclosure of sexual deviance. Psychotherapy.

[60] Schneider JP, Schneider BH (1996). Couple recovery from sexual addiction/coaddiction: Results of a survey of 88 marriages. Sexual Addiction & Compulsivity.

[61] Ravart M, Trudel G, Marchand A, Turgeon L (1996). The efficacy of a cognitive behavioural treatment model for hypoactive sexual desire disorder: An outcome study. Can J Hum Sex.

[62] Hurlbert DF, White LC, Powell RD, Apt C (1993). Orgasm consistency training in the treatment of women reporting hypoactive sexual desire: an outcome comparison of women-only groups and couples-only groups. J BehavTherExp Psychiatry.

[63] Leszcz M, Goodwin PJ (1998). The rationale and foundations of group psychotherapy for women with metastatic breast cancer. Int J Group Psychother.

[64] Barlow SH, Burlingame GM, Fuhriman A (2000). Therapeutic applications of groups: From Pratt’s “thought control classes” to modern group psychotherapy. Group Dyn.

[65] Walker BL (1997). Adjustment of husbands and wives to breast cancer. Cancer Pract.

[66] McRoberts C, Burlingame GM, Hoag MJ (1998). Comparative efficacy of individual and group psychotherapy: A meta-analytic perspective. Group Dyn.

[67] Zarcone J, Smithline L, Koopman C, Kraemer HC, Spiegel D (1995). Sexuality and spousal support among women with advanced breast cancer. Breast J.

[68] Kalaitzi C, Papadopoulos VP, Michas K, Vlasis K, Skandalakis P (2007). Combined brief psychosexual intervention after mastectomy: effects on sexuality, body image, and psychological well-being. J SurgOncol.

[69] Classen C, Diamond S, Spiegel D, VandeCreek L, Jackson TL, Sarasota FL (1999). Supportive-expressive group therapy for cancer and HIV patients. Innovations in clinical practice: A source book.

[70] Yalom ID (1980). Existential Psychotherapy.

[71] Yalom ID, Greaves C (1977). Group therapy with the terminally ill. Am J Psychiatry.

[72] Yalom ID, Leszcz M (2005). The Theory and Practice of Group Psychotherapy.

[73] Passik SD, Wilson A (1987). Technical issues on the frontier between supportive and expressive modes in psychotherapy. DynamPsychother.

[74] Spiegel D (1993). Living Beyond Limits: New Hope and Help for Facing Life-Threatening Illness.

[75] Spiegel D (1995). Essentials of psychotherapeutic intervention for cancer patients. Support Care Cancer.

[76] Spiegel D, Classen C (2000). Group Therapy for Cancer Patients: A research-Based Handbook of Psychosocial Care.

[77] Spiegel D, Morrow GR, Classen C, Raubertas R, Stott PB (1999). Group psychotherapy for recently diagnosed breast cancer patients: A multicenter feasibility study. Psychooncology.

[78] Classen C, Abramson S, Angell K, Atkinson A (1997). Effectiveness of a training program for enhancing therapists’ understanding of the supportive-expressive treatment model for breast cancer groups. J PsychotherPract Res.

[79] Reuter K, Scholl I, Sillem M, Hasenburg A, Harter M (2010). Implementation and Benefits of Psychooncological Group Interventions in German Breast Centers: A Pilot Study on Supportive-Expressive Group Therapy for Women with Primary Breast Cancer. Breast Care (Basel).

[80] Spiegel D (1994). Health caring. Psychosocial support for patients with cancer. Cancer.

[81] Spiegel D, Bloom JR, Kraemer HC, Gottheil E (1989). Effect of psychosocial treatment on survival of patients with metastatic breast cancer. Lancet.

[82] Classen C, Diamond S, Soleman A, Fobair P, Spira J (1993). Brief supportive-expressive group therapy for women with primary breast cancer: A treatment manual.

[83] Diamond S, Gore-Felton C, Gale AG, Classen C, Spiegel D (1996). Supportive-Expressive group therapy for persons with HIV infection: A treatment manual.

[84] Spiegel D, Spira J (1991). Supportive/Expressive Group Therapy: A treatment manual of psychosocial intervention for women with recurrent breast cancer.

[85] Classen C, Butler LD, Koopman C, Miller E, DiMiceli S (2001). Supportive-expressive group therapy and distress in patients with metastatic breast cancer: a randomized clinical intervention trial. Arch Gen Psychiatry.

[86] Spiegel D, Morrow GR, Classen C, Raubertas R, Stott PB (1999). Group psychotherapy for recently diagnosed breast cancer patients: a multicenter feasibility study. Psychooncology.

[87] Butler LD, Koopman C, Neri E, Giese-Davis J, Palesh O (2009). Effects of supportive-expressive group therapy on pain in women with metastatic breast cancer. Health Psychol.

[88] Spiegel D, Bloom JR (1983). Group therapy and hypnosis reduce metastatic breast carcinoma pain. Psychosom Med.

[89] Spiegel D, Butler LD, Giese-Davis J, Koopman C, Miller E (2007). Effects of supportive-expressive group therapy on survival of patients with metastatic breast cancer: a randomized prospective trial. Cancer.

[90] Spiegel D (2011). Mind matters in cancer survival. JAMA.

[91] Kelly JA, Murphy DA, Bahr GR, Kalichman SC, Morgan MG (1993). Outcome of cognitive-behavioral and support group brief therapies for depressed, HIV-infected persons. Am J Psychiatry.

[92] Lutgendorf SK, Sood AK, Anderson B, McGinn S, Maiseri H (2005). Social support, psychological distress, and natural killer cell activity in ovarian cancer. J ClinOncol.

[93] Koopman C, Hermanson K, Diamond S, Angell K, Spiegel D (1998). Social support, life stress, pain and emotional adjustment to advanced breast cancer. Psychooncology.

[94] Butler LD, Koopman C, Classen C, Spiegel D (1999). Traumatic stress, life events, and emotional support in women with metastatic breast cancer: Cancer-related traumatic stress symptoms associated with past and current stressors. Health Psychol.

[95] Baider L, Ever-Hadani P, Goldzweig G, Wygoda MR, Peretz T (2003). Is perceived family support a relevant variable in psychological distress?. A sample of prostate and breast cancer couples. J Psychosom Res.

[96] Mulder C, van der Pompe G, Spiegel D, Antoni M (1992). Do psychosocial factors influence the course of breast cancer? A review of recent literature, methodological problems and future directions. Psycho-Oncology.

[97] Marmarosh CL, Corazzini JG (1997). Putting the group in your pocket: Using collective identity to enhance personal and collective self-esteem. Group Dyn.

[98] Manne SL, Alfieri T, Taylor KL, Dougherty J (1999). Spousal negative responses to cancer patients: the role of social restriction, spouse mood, and relationship satisfaction. J Consult ClinPsychol.

[99] Giese-Davis J, Sephton SE, Spiegel D (2000). Repression associated with a physiological risk factor for shorter survival in women with metastatic breast cancer.

[100] Giese-Davis J, Spiegel D (2001). Emotional expression, diurnal cortisol, and survival.

[101] Greer S (1991). Psychological response to cancer and survival. Psychol Med.

[102] Classen C, Koopman C, Angell K, Spiegel D (1996). Coping styles associated with psychological adjustment to advanced breast cancer. Health Psychol.

[103] Greer S, Moorey S, Baruch JD, Watson M, Robertson BM (1992). Adjuvant psychological therapy for patients with cancer: a prospective randomised trial. BMJ.

[104] Giese-Davis J, Koopman C, Butler L, Classen C, Cordova M (2002). Change in emotion-regulation strategy for women with metastatic breast cancer following supportive-expressive group therapy. J Consult ClinPsychol.

[105] Spiegel D, Glafkides MC (1983). Effects of group confrontation with death and dying. Int J Group Psychother.

[106] Weiner DN, Rosen RC, Sipski ML, Alexander CJ (1997). Medications and their impact. Sexual function in people with disability and chronic illness: a health professional’s guide.

[107] Giese-Davis J, Hermanson K, Koopman C, Weibel D, Spiegel D (2000). Quality of couples’ relationship and adjustment to metastatic breast cancer. J FamPsychol.

[108] Chochinov HM, Kristjanson LJ, Breitbart W, McClement S, Hack TF (2011). Effect of dignity therapy on distress and end-of-life experience in terminally ill patients: a randomised controlled trial. Lancet Oncol.

[109] Yalom ID (2008). Staring at the Sun.

[110] Spiegel D (1990). Facilitating emotional coping during treatment. Cancer.

[111] Spiegel D, Bloom JR, Gottheil E (1983). Family Environment as a Predictor of Adjustment to Metastatic Breast Carcinoma. J PsychosocOncol.

[112] Jarrett SR, Ramirez AJ, Richards MA, Weinman J (1992). Measuring coping in breast cancer. J Psychosom Res.

[113] Bodenmann G, Ledermann T, Blattner D, Galluzzo C (2006). Associations among everyday stress, critical life events, and sexual problems. J NervMent Dis.

[114] Grassi L, Sabato S, Rossi E, Marmai L, Biancosino B (2010). Effects of supportive-expressive group therapy in breast cancer patients with affective disorders: a pilot study. PsychotherPsychosom.

[115] Cull A, Cowie VJ, Farquharson DI, Livingstone JR, Smart GE (1993). Early stage cervical cancer: psychosocial and sexual outcomes of treatment. Br J Cancer.

[116] Ghizzani A, Pirtoli L, Bellezza A, Velicogna F (1995). The evaluation of some factors influencing the sexual life of women affected by breast cancer. J Sex Marital Ther.

[117] Sacerdoti RC, Lagana’ L, Koopman C (2010). Altered Sexuality and Body Image after Gynecological Cancer Treatment: How Can Psychologists Help?. Prof Psychol Res Pr.

[118] Fallowfield LJ, Hall A (1991). Psychosocial and sexual impact of diagnosis and treatment of breast cancer. Br Med Bull.

[119] Fobair P, Spiegel D (2009). Concerns about sexuality after breast cancer. Cancer J.

[120] Braun M, Mikulincer M, Rydall A, Walsh A, Rodin G (2007). Hidden morbidity in cancer: spouse caregivers. J ClinOncol.

[121] Scott JL, Halford WK, Ward BG (2004). United we stand? The effects of a couple-coping intervention on adjustment to early stage breast or gynecological cancer. J Consult ClinPsychol.

[122] Lagana L, Classen C, Caldwell R, McGarvey E, Baum L (2005). Sexual difficulties of patients with gynecological cancer. Prof Psychol-Res Pr.

[123] Lagana L, McGarvey EL, Classen C, Koopman C (2001). Psychosexual dysfunction among gynecological cancer survivors. J ClinPsychol Med S.

[124] Schover LR (1991). The impact of breast cancer on sexuality, body image, and intimate relationships. CA Cancer J Clin.

[125] Spencer SM, Lehman JM, Wynings C, Arena P, Carver CS (1999). Concerns about breast cancer and relations to psychosocial well-being in a multiethnic sample of early-stage patients. Health Psychol.

[126] Schover LR, Rhodes MM, Baum G, Adams JH, Jenkins R (2011). Sisters Peer Counseling in Reproductive Issues After Treatment (SPIRIT): a peer counseling program to improve reproductive health among African American breast cancer survivors. Cancer.

[127] Taylor S, Harley C, Ziegler L, Brown J, Velikova G (2011). Interventions for sexual problems following treatment for breast cancer: a systematic review. Breast Cancer Res Treat.

